# Prenatal exposure to diethylstilbestrol has multigenerational effects on folliculogenesis

**DOI:** 10.1038/s41598-024-81093-8

**Published:** 2024-12-28

**Authors:** Rachael E. Rogers, Kate A. Fowler, Andrew J. Pask, Deidre M. Mattiske

**Affiliations:** School of BioSciences, The University of Melbourne, Melbourne, 3010 Australia

**Keywords:** Infertility, Reproductive disorders

## Abstract

Diethylstilbestrol (DES) is an estrogenic endocrine disrupting chemical (EDC) that was prescribed to millions of pregnant women worldwide, leading to increased rates of infertility in the exposed offspring. We have previously demonstrated that this reduced fertility persists for multiple generations in the mouse. However, how altered ovarian function contributes to this infertility is unknown. Therefore, this study sought to determine if DES exposure promotes two common ovarian disorders, primary ovarian insufficiency (POI) and polycystic ovary syndrome, contributing to the reduced fertility in DES offspring. Moreover, we investigated if these impacts are transgenerational. Gestating mice were exposed to 100 µg/kg DES, and ovarian morphology was observed in F1-F3 female descendants. F1 females trended towards fewer primordial and more secondary follicles and similarly, F2 females had fewer primordial and significantly more secondary follicles compared to controls. No differences in follicle proportions were observed in the F3. Moreover, DES exposure did not increase follicular cysts. These results show that DES accelerates folliculogenesis, indicative of a POI phenotype and that this is likely contributing to the reduced fertility observed in DES descendants. Moreover, this study highlights the ability of estrogenic EDCs to disrupt folliculogenesis, which may exacerbate the onset of POI in women already at risk.

## Introduction

Infertility is one of the most pressing issues affecting society today, with 15% of reproductive aged couples experiencing infertility in their lifetime^1^. A major contributor to this increasing rate of infertility is our exposure to endocrine disrupting chemicals (EDCs). We are continuously exposed to EDCs in our everyday life as they are present in plastics, cosmetics, pharmaceuticals, herbicides, and pesticides. Although exposure to EDCs is harmful during all stages of life, developmental exposure to EDCs poses the greatest threat as EDCs can alter the fetal hormonal environment through disrupting normal hormonal actions, resulting in an increased risk of developing reproductive abnormalities later in life^2^.

The estrogenic EDC diethylstilbestrol (DES) provides a clear example of the adverse effects early life EDC exposure can have on reproductive development^3^. DES is pharmaceutical drug that was prescribed to millions of pregnant women world-wide in the hopes to prevent miscarriage and premature labour, however, it resulted in detrimental reproductive effects in the offspring exposed^3^. Specifically, DES daughters have increased rates of reproductive tract tumors and pregnancy complications, including preterm birth, miscarriage, and ectopic pregnancy, as well as increased rates of infertility, at 33% compared to 14% in unexposed women^4,5^. Similar abnormalities have been described in the DES mouse model, with prenatal exposure resulting in structural malformations of the oviduct, uterus, cervix and vagina^6^. High rates of infertility have also been described in the DES mouse model^7,8^. However, despite this increased infertility observed in both DES daughters and animals, few studies have investigated the effect of DES on folliculogenesis and if abnormal ovarian function contributes to this reduced fertility.

Females reproductive capacity relies on correct ovarian function, specifically, folliculogenesis and subsequent ovulation^9^. Folliculogenesis is the process by which primordial follicles are recruited from the resting pool and mature until they are either ovulated or die via atresia^9^. In humans, primordial follicle formation begins by week 20 of gestation and the pool that is formed represents all the follicles that will be accessible to a female over the course of her reproductive life^10^. After primordial follicle formation, a subset are selected to grow and mature into primary follicles and continue to progress into antral follicles, while the remaining follicles stay dormant^11^. This process is known as initial recruitment and occurs in a continuous manner throughout a females reproductive lifespan^12^. After the onset of puberty, a subset of antral follicles are rescued from atresia due to an increase in circulating follicle stimulating hormone (FSH). These antral follicles continue to develop and mature, with one follicle becoming dominant and producing high levels of estrogen and inhibins^12^. This increase in estrogen exerts a negative feedback loop and suppresses FSH secretion such that the remaining growing antral follicles are deprived of adequate FSH stimulation required for survival and die via atresia. Due to the increase in estrogen, luteinising hormone is released and ovulation of the dominant follicle is triggered^12^. This process is known as cyclic recruitment and occurs every month until the follicle pool is depleted, leading to reproductive senescence. Therefore exposure to EDCs, like DES, could impact initial and cyclic recruitment, leading to altered folliculogenesis and subsequent infertility.

Two common ovarian disorders that impact folliculogenesis and can contribute to infertility are primary ovarian insufficiency (POI) and polycystic ovary syndrome (PCOS). POI affects 1% of reproductive aged women and disrupts ovarian function such that menopause is reached before the age of 40^13^. This early decline in ovarian function can be due to an exhaustion of the primordial follicle pool, increased activation of primordial follicles, increased follicle atresia or disrupted ovulation^14^. The aetiology of POI is largely unknown, with genetic causes being responsible for only 10.8% of POI cases^15^. Environmental factors are able to contribute to POI, with exposure to EDCs significantly impacting the ovarian reserve and increasing the risk of developing POI^14^. Female mice exposed neonatally to 20 µg/kg of DEHP, an EDC with both estrogenic and anti-androgenic properties, have significantly fewer primordial follicles and significantly more pre-antral follicles, indicating accelerated folliculogenesis^16^. Furthermore, prenatal exposure to a mixture of phthalates significantly reduces the number of secondary follicles and significantly increases the number of atretic follicles in PND 21 and 9 month old female mice^17^. Human studies have demonstrated the impacts of the estrogenic EDC BPA on folliculogenesis, with high levels of urinary BPA being associated with decreased antral follicle counts^18^.

PCOS is one of the most common reproductive diseases in women, affecting approximately 5–15% of the female population^19^. Symptoms of PCOS include increased androgen levels, abnormal ovulation and cystic ovaries. Long term impacts of PCOS include infertility, type 2 diabetes and cardiovascular disease^20^. Currently the aetiology of PCOS involves both genetic and environmental factors, with exposure to EDCs playing a role in disease prevalence^21^. Notably, women with PCOS have significantly higher levels of BPA in their blood compared to women without PCOS^22^. Moreover, rats exposed developmentally to a mixture of EDCs results in an increased number of cystic ovaries^23^.

EDCs impact many aspects of ovarian development, however, of greatest concern is their ability cause long-lasting, transgenerational effects on the ovary. Transgenerational effects refer to inherited disorders that are observed in individuals that have not been directly exposed. When a gestating female is exposed to an EDC, herself, the F1 fetus and the germ cells that will produce the F2 generation are all directly exposed. Therefore, any effects seen in these generations are known as multigenerational and are due to a direct exposure. The F3 generation however, is the first generation that hasn’t had any exposure to the EDC and effects observed in the F3 and beyond are known as transgenerational. Transgenerational inheritance of ovarian disease has been described in animal models after exposure to several EDCs. Neonatal exposure to DEHP significantly decreases the number of primordial follicles in F1, F2 and F3 female mice whilst prenatal exposure to both a mixture of EDCs and methoxychlor increases the rate of cystic ovaries in F3 female rats^23–25^. We have previously shown that exposure to DES has transgenerational effects on fertility, timing of puberty and the anogenital distance (AGD) in female DES descendants^8^. However, the transgenerational effect of DES on the ovary has yet to be investigated.

Therefore, the aim of this study was to determine if DES exposure alters folliculogenesis and whether this can contribute to the reduced fertility observed in the DES mouse model. Additionally, it sought to assess whether DES exposure has a transgenerational effect on the ovary. The transgenerational effects caused by EDCs is particularly alarming for the more than 50 million DES descendants, as they may have a higher risk of experiencing infertility and other reproductive abnormalities due to their ancestral exposure^26,27^. Moreover, the results gathered from this study can be used as a model for other estrogenic EDCs that are pervasive in our environment and deepen our understanding of the transgenerational effects EDCs.

## Results

### Young cohort

#### DES does not impact female body weight and AGD during puberty

**Fig. 1:**
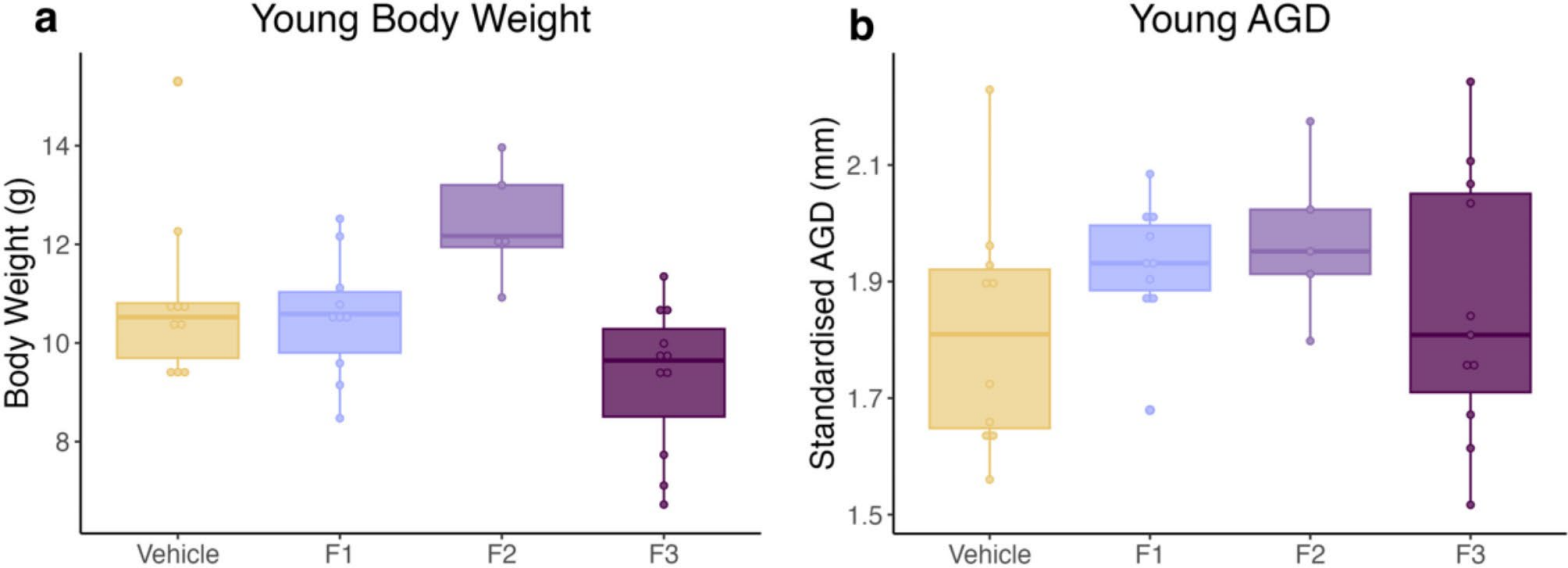
Body weight and AGD of young cohort. **(a)** DES exposure did not have any significant effect on body weight or **(b)** standardised AGD in the young cohort in any generation. (*n* = 10, 10, 5 and 11 for vehicle, F1, F2 and F3 respectively).

Previous animal studies have demonstrated an increase in body weight after exposure to EDCs due to their ability to impair metabolic function^23,28^. In this study, we found that DES exposure did not significantly impact body weight at PND 21 in any generation compared to the control cohort. AGD is used to monitor sexual differentiation and as a marker of the hormonal environment in which the embryo developed. To account for body size effects on AGD, AGD was standardised by dividing by the cubed root of body weight (AGD/$\:\sqrt[3]{BW}$)^29^. Prenatal DES exposure did not have any significant effect on standardised AGD in the young cohort at any generation compared to control females (Fig. 1).

#### DES impacts folliculogenesis in a multigenerational manner

**Fig. 2:**
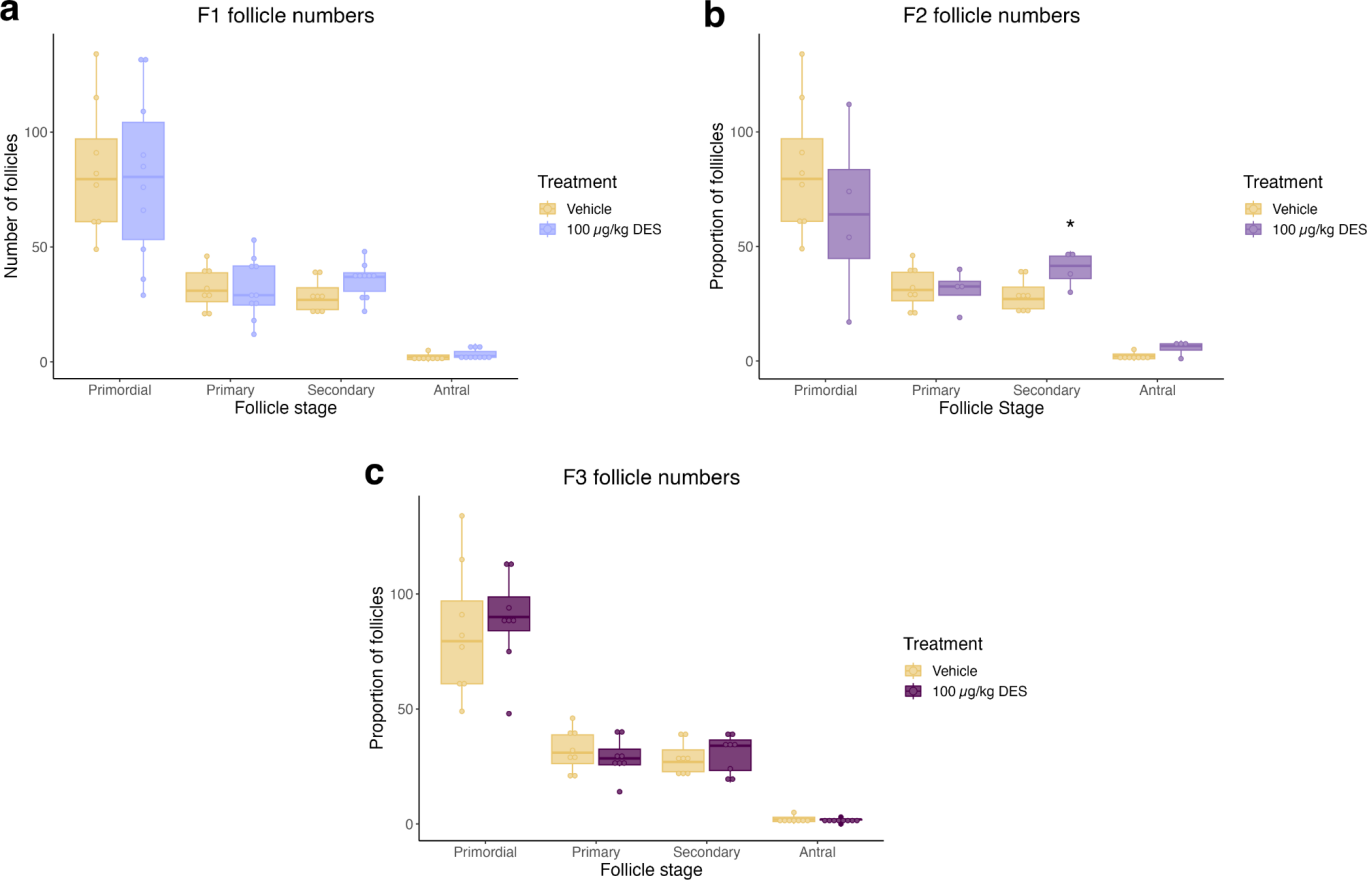
Effect of prenatal DES exposure on follicle numbers. Ovaries were collected from PND21 control and F1-F3 DES treated females and serially sectioned at 7 μm. Every 10th section was used to count primordial, primary, secondary and antral follicles. **a)** The F1 generation trended towards more secondary follicles compared to the control. **(b)** DES significantly increased the number of secondary follicles in the F2 generation. **(c)** No significant difference in follicle numbers were observed in the F3 generation. (* *p* < .05) (*n* = 8, 10, 4 and 8 for vehicle, F1, F2 and F3 respectively).

To determine if DES exposure can lead to a POI phenotype, follicle counts were conducted on PND 21 ovaries in control and F1-F3 females. Results are presented as both total follicle number and follicle proportions. DES did not have a significant effect on follicle numbers in the F1 generation, however, a trend towards more secondary follicles was observed (Fig. 2). In the F2 generation, DES exposure significantly increased the number of secondary follicles and although not significant, the median number of primary follicles was reduced (Fig. 2, *p* = .050). DES did not have a significant impact on the number of follicles in the F3 generation (Fig. 2).

**Fig. 3:**
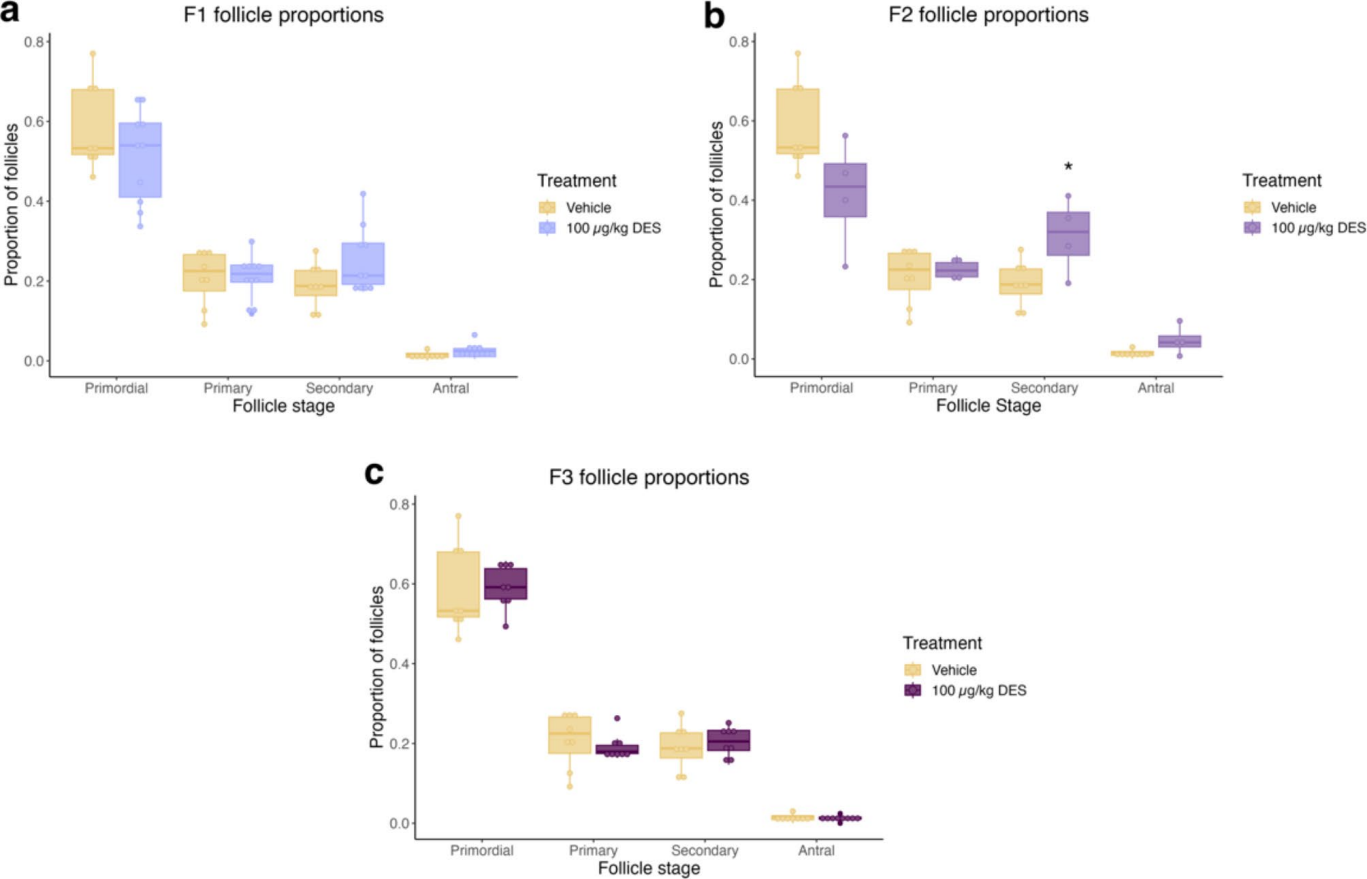
Effect of prenatal DES exposure on proportion of follicle types. Follicle types represented as a proportion of the total number of healthy follicles. **(a)** The F1 generation trended towards fewer primordial and more secondary follicles compared to the control. **(b)** DES significantly increased the proportion of secondary follicles in the F2 generation. **(c)** No significant difference in follicle proportions were observed in the F3 generation. (* *p* < .05) (*n* = 8, 10, 4 and 8 for vehicle, F1, F2 and F3 respectively).

**Fig. 4:**
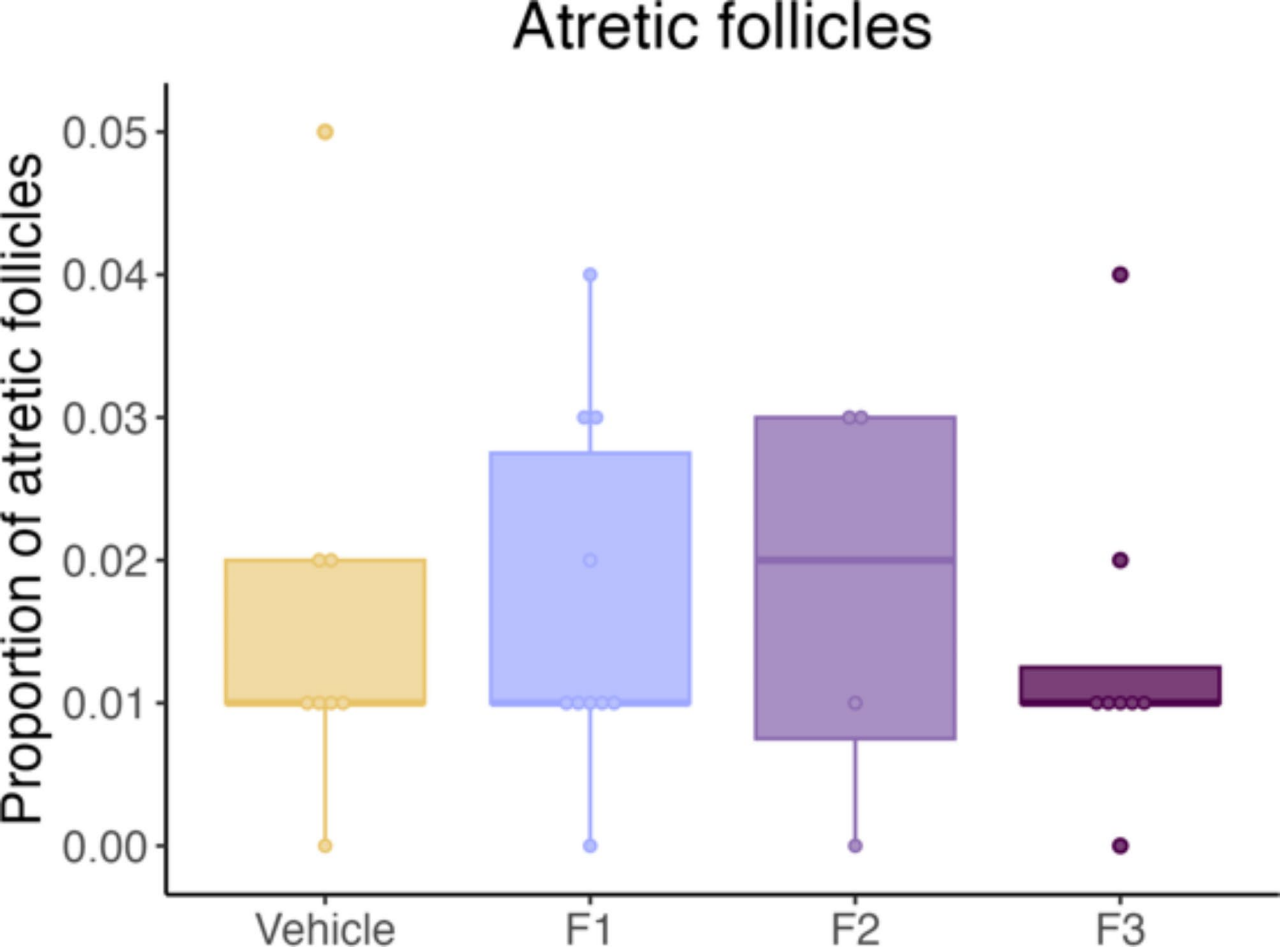
Proportion of atretic follicles in young cohort. Follicles were defined as atretic if two or more of the following features were present: degenerating oocyte, GC layer containing pyknotic nuclei, detached GC layer from the basement membrane or a broken basement membrane. DES exposure had no significant effect on the proportion of atretic follicles in any generation.

To account for differences in the total number of follicles between individuals, follicle types were presented as proportions relative to the overall number of healthy follicles. Similar to total follicle numbers, DES did not have a significant effect on the proportion of follicle types in the F1 generation, however a trend towards fewer primordial and more secondary follicles was observed (Fig. 3). DES exposure significantly increased the number of secondary follicles in the F2 generation and moreover, the median proportion of primordial follicles is reduced compared to the control (Fig. 3, *p* = .048). DES did not have any significant impact on any follicle proportions in the F3 generation (Fig. 3). No significant difference in the proportion of atretic follicles were observed between any of the DES generations and the control cohort (Fig. 4).

#### DES does not impact ovarian somatic cell morphology, apoptosis, or proliferation

**Fig. 5:**
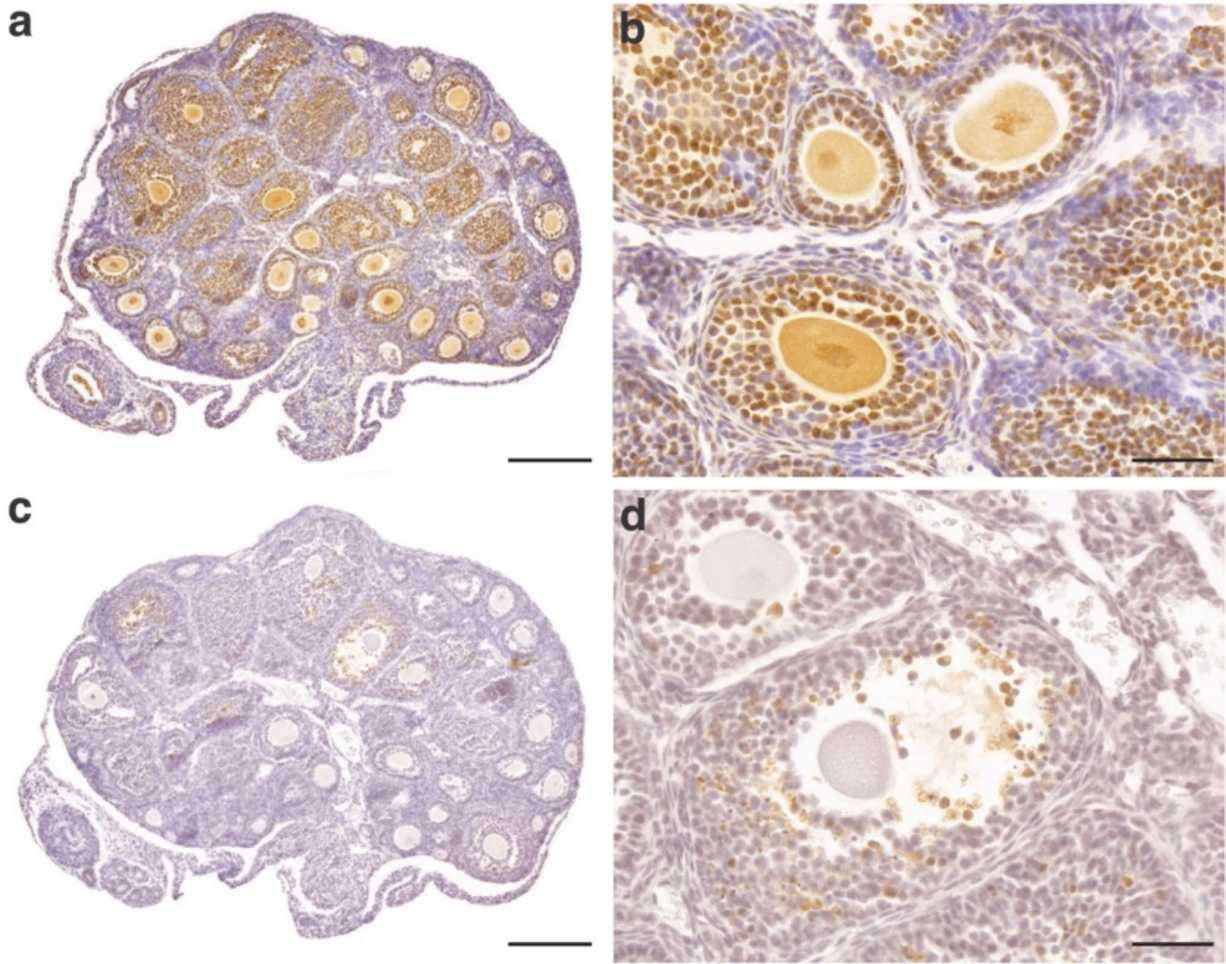
IHC sections of PND 21 control ovaries.** (a)** and **(b)** are representative images of PCNA stained ovaries. **(c)** and **(d)** are representative images of Casp3 stained ovaries. **(a)** and **(c)** scale bar = 200 μm, **(b)** and **(d)** scale bar = 50 μm.

**Fig. 6:**
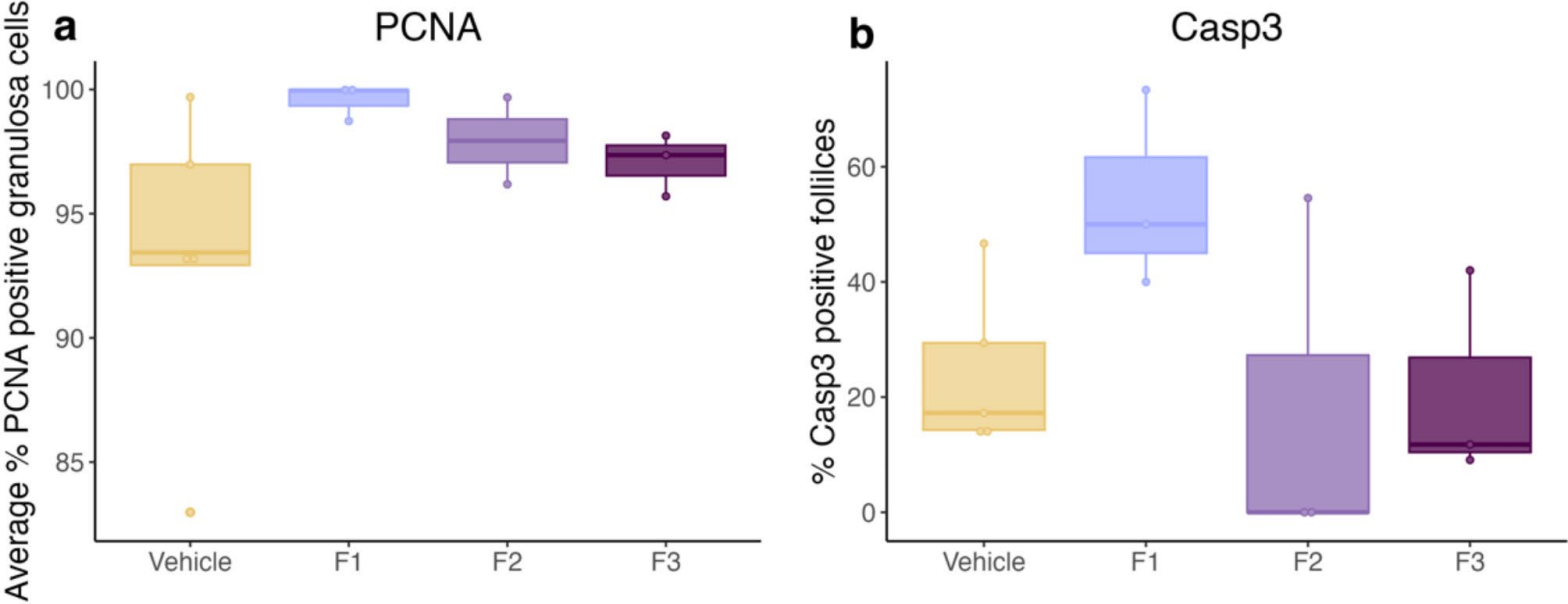
GC proliferation and apoptosis was analysed via PCNA and Casp3 antibodies respectively.** (a)** DES exposure had no significant effect on GC proliferation across any generation. **(b)** DES increased the median percentage of Casp3 positive follicles in the F1 generation, however this was not significant. (PCNA, *n* = 5, 3, 2, 3 and Casp3, *n* = 5, 3, 3, 3 for vehicle, F1, F2 and F3 respectively).

Granulosa cell (GC) proliferation and apoptosis was marked using proliferating cell nuclear antigen (PCNA) and cleaved caspase-3 (Casp3) primary antibodies respectively (Fig. 5). PCNA positive GCs were calculated as a percentage of the total number GCs within a follicle. No significant difference in the percentage of proliferating cells was observed between DES generations and the control (Fig. 6). Atretic follicles were defined as those with 5% or greater Casp3 positive cells. Prenatal DES exposure increased the median percentage of atretic follicles in the F1 generation, however this was not significant. No difference in atretic follicles was observed in the F2 or F3 generation (Fig. 6).

**Fig. 7:**
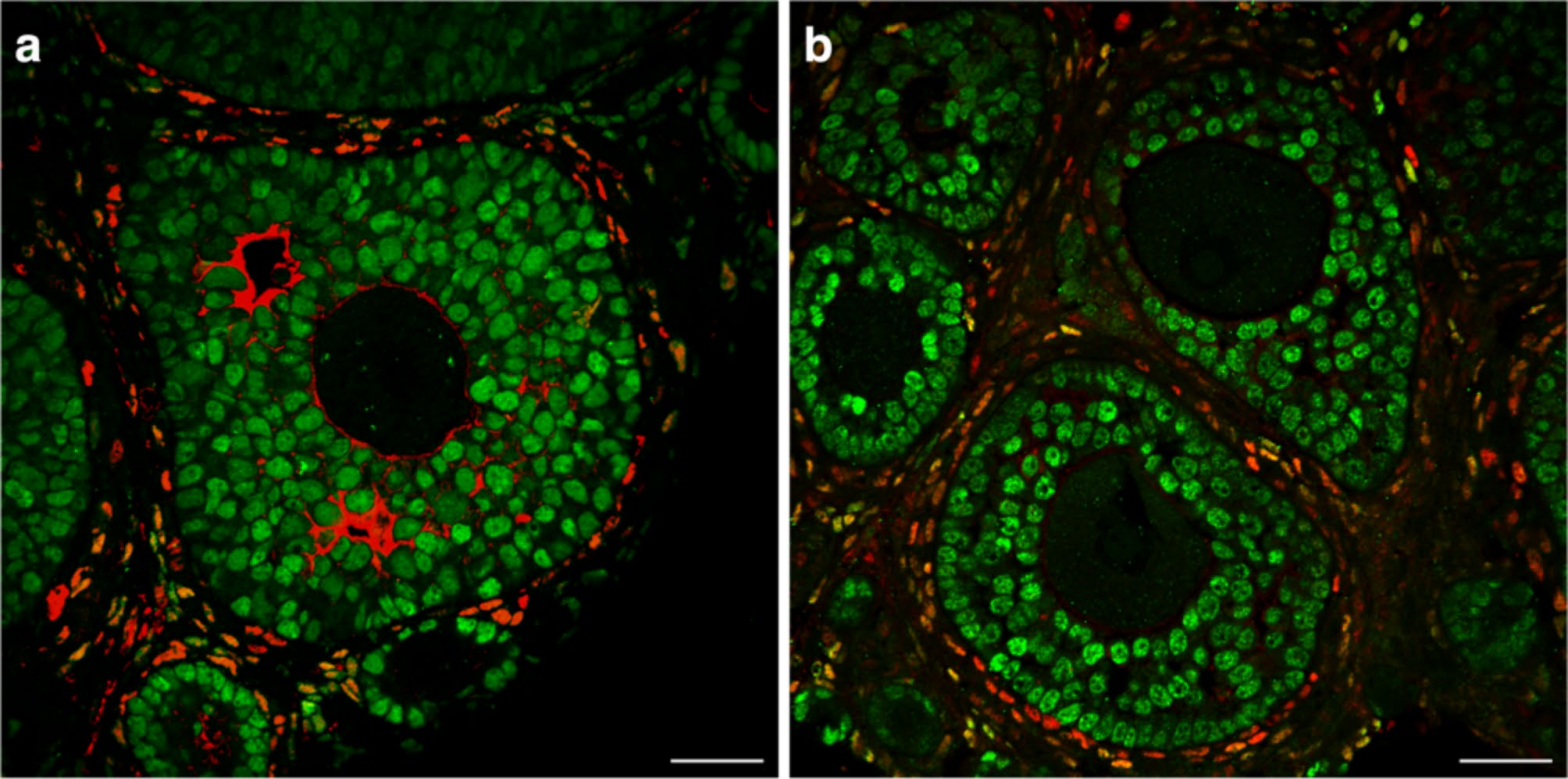
IF sections of PND 21 ovaries. No difference in GC (FOXL2; green) or theca cell (COUP-TFII; red) morphology was observed. **(a)** represents a control ovary; **(b)** represents a F1 DES treated ovary. Scale bar = 25 μm.

Immunofluorescence (IF) was used to analyse granulosa and theca cell morphology. Representative sections from the middle portion of the ovary were used for analysis. No significant morphological differences were observed in both granulosa and theca cells. GCs appeared cuboidal in shape and were evenly dispersed throughout the follicle in both control and DES ovaries. Similarly, theca cells formed distinct externa and interna layers around the ovarian follicles in control and DES ovaries (Fig. 7).

### Aged cohort

#### DES does not impact body weight but reduces AGD in aged females

**Fig. 8:**
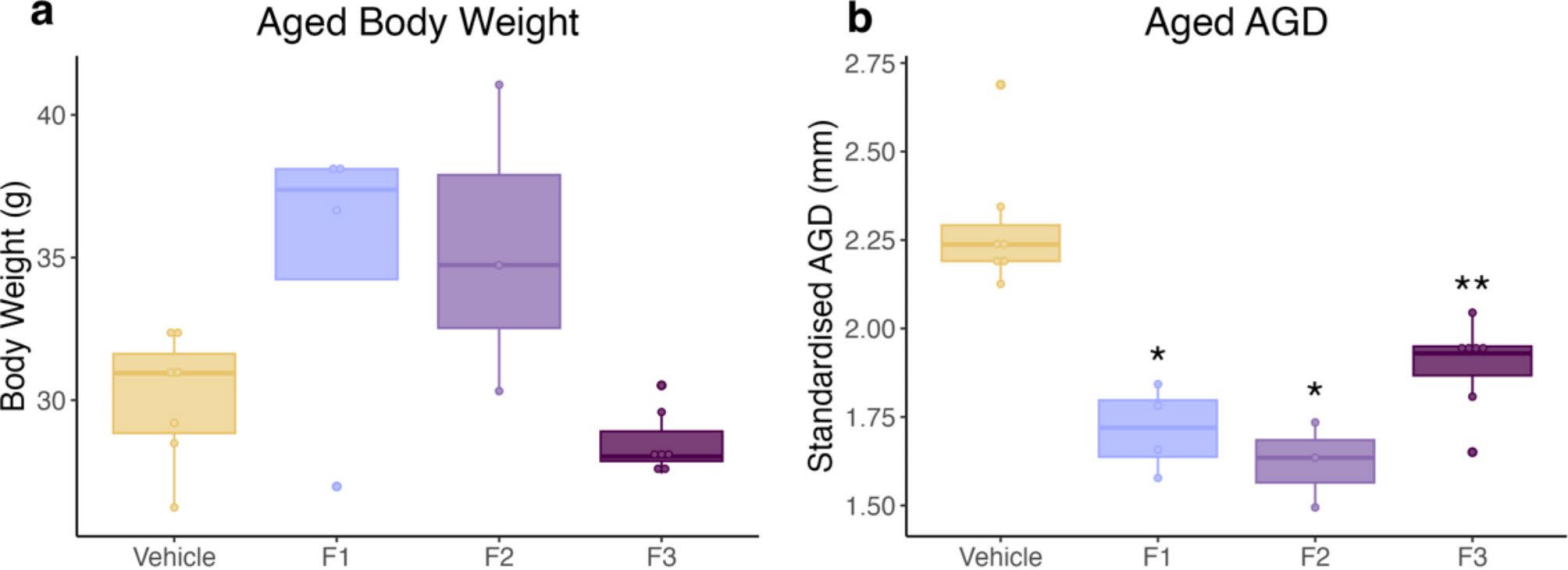
Body weight and AGD of aged cohort.** (a)** Median body weight was increased in F1 and F2 DES females, although this increase was not significant. **(b)** DES exposure significantly reduced the standardised AGD in the F1, F2 and F3 generation. (* *p* < .05; ** *p* < .01) (*n* = 7, 4, 3, 7 respectively).

DES exposure increased the median body weight in F1 and F2 females, although this was not significant. No differences in body weight was observed in the F3 generation (Fig. 8). DES exposure significantly reduced standardised AGD in the F1, F2 and F3 generations compared to the control cohort (Fig. 8, *p* = 0.0182, 0.0333 and 0.0035 respectively).

#### DES does not promote a PCOS phenotype

**Fig. 9:**
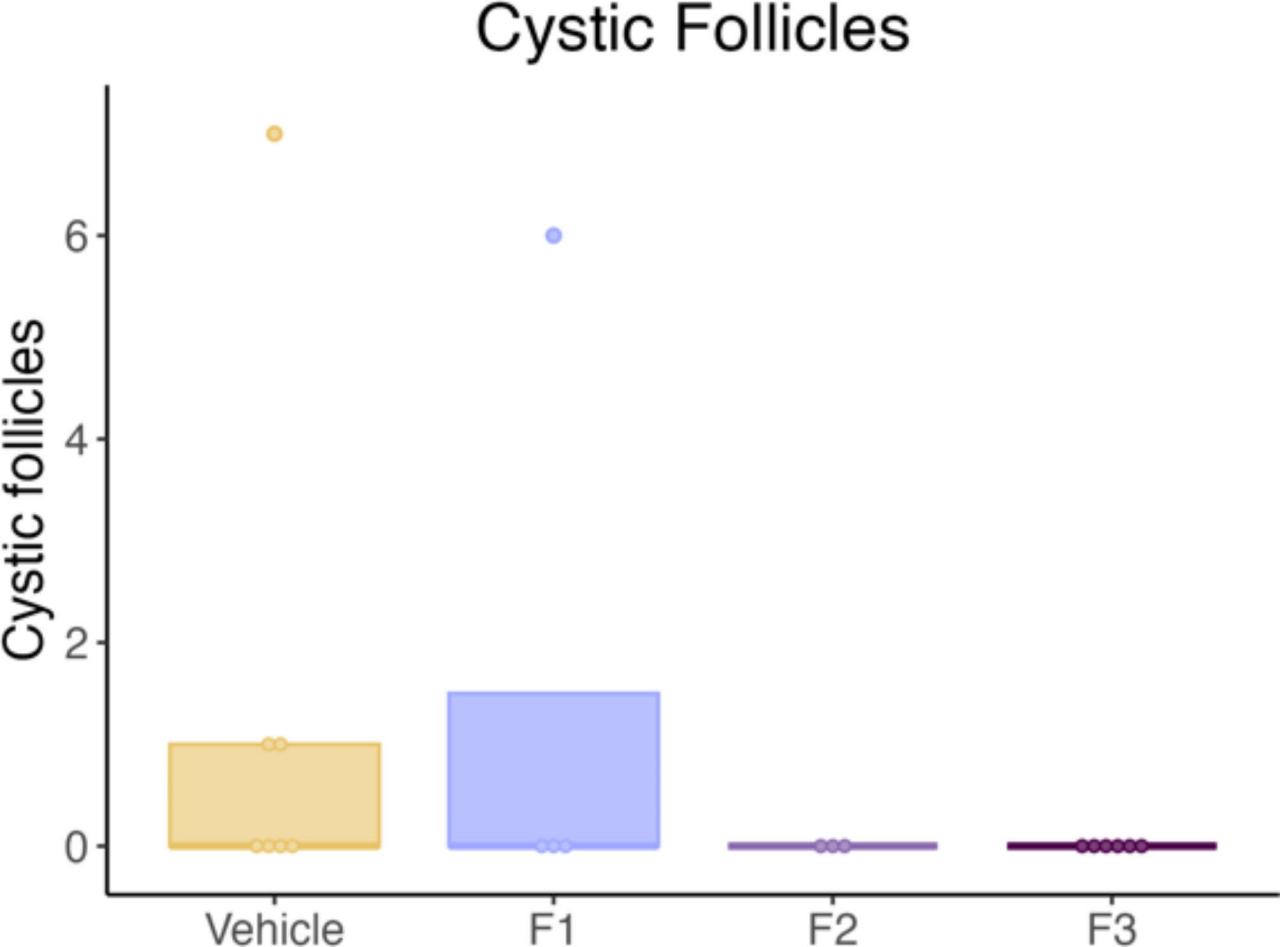
Number of cystic follicles in aged cohort. A follicular cyst was defined as a fluid-filled structure lacking GCs and an oocyte and lined by one to four layers of cuboidal GCs. DES exposure had no significant effect on the number of cystic follicles.

Ovaries collected from the aged cohort were used to observe the presence of follicular cysts to determine if DES exposure is able to promote a PCOS phenotype. Follicular cysts were only observed in control and F1 DES females and there was no significant difference in the proportion of follicular cysts between these groups (Fig. 9).

## Discussion

Infertility is a growing issue in society today with exposure to EDCs playing a major role. One way in which EDCs can contribute to female infertility is through altering ovarian development and function. We have previously shown that exposing gestating mice to a dose of 100 µg/kg of DES reduces females fertility in a multigenerational manner^8^. Our results are consistent with data from DES daughters, who experience higher rates of infertility compared to unexposed women^5^. Therefore, we sought to determine if DES exposure could promote two ovarian diseases that disrupt folliculogenesis, POI and PCOS, and therefore contribute to the observed infertility in these descendants. Furthermore, we investigated if DES exposure has a transgenerational effect on these phenotypes. We found that DES does not impact the number of follicular cysts, however, it does impact folliculogenesis in F1 and F2 females. These results indicate that a direct exposure to 100 µg/kg of DES can contribute to the onset of POI which in turn may contribute to the reduced fertility observed after prenatal DES exposure.

EDCs are able to interfere with hormones involved in metabolism and promote weight gain and obesity with prenatal exposure to DES, and other EDCs, increasing body weight in adult female mice^30–32^. However, our study found that *in utero *exposure to DES does not have an effect on body weight in PND 21 females but the median body weight is increased in 12 month old F1 and F2 DES females. We have previously shown that median body weight is increased in 5 month old females after exposure to 100 µg/kg of DES, and that this increase is greater in F1 and F2 females compared to F3 females^8^. Therefore, a direct exposure to DES promotes a small increase in body weight once a female is sexually mature, not only in F1 females that are exposed as a fetus, but also in F2 females that are only exposed as a germ cell, but this effect is not inherited transgenerationally.

It is surprising that DES exposure did not impact the AGD in the young cohort but did significantly reduce the AGD in all three generations in the aged cohort. We have previously shown that 100 µg/kg of DES significantly reduces the AGD in F1, F2 and F3 females at 5 months of age^8^. The AGD is a key marker for reproductive toxicity, with the development of the AGD being highly sensitive to the fetal hormonal environment. As we have seen a significant reduction in the AGD in post-pubertal but not pre-pubertal females, this suggests that DES exposure is likely having a greater impact on the maturation rather than on the early patterning and differentiation of the AGD. Sexual maturation and the development of the female external genitalia continues postnatally and is programmed by hormonal exposures during fetal development. Furthermore, the transgenerational impact seen on the AGD, but not folliculogenesis, indicates that DES is disrupting hormonal signalling pathways specific to AGD development in a transgenerational manner.

To determine if altered folliculogenesis can contribute to the reduced fertility observed in DES descendants, we quantified the proportion of follicle types in PND 21 females. DES exposure significantly increased the proportion of secondary follicles in F2 females, and although not significant, the median proportion of primordial follicles was reduced compared to the control cohort. This reduction in primordial follicles and increase in secondary follicles suggests follicle recruitment is accelerated in the F2 DES generation leading to early exhaustion of the resting follicle pool. The F1 generation displayed a similar acceleration of folliculogenesis, although not statistically significant, with a trend towards fewer primordial and more secondary follicles being observed. Interestingly, this trend was not observed in the F3 generation in our study, indicating that this effect is not transgenerational at a dose of 100 µg/kg of DES and only occurs in individuals that are exposed either during embryogenesis or during initial primordial germ cell development.

A similar acceleration of folliculogenesis is observed in F1 mice after prenatal exposure to a single injection of DES on gestation day 15, with later stage follicles being observed 24 h earlier in treatment animals compared to controls^33^. In contrast, McLachlan et al. found no significant difference in follicle proportions in PND 60 mice after prenatal exposure to doses of DES ranging from 0.01 to 100 µg/kg^7^. However, primordial and primary follicles were classified as one follicle type in this study and therefore, if the now standardised classification of follicle types was applied, effects may be observed. Interestingly, both rats and lambs treated neonatally with either DES or BPA have significantly fewer primordial and significantly more primary follicles compared to control ovaries, similar to the effects observed in our study^34,35^. In contrast, mice neonatally exposed to 3 µg of DES on PND 0 and PND 1 results in significantly more primordial follicles and significantly fewer primary follicles by PND 5, suggesting that neonatal DES exposure delays follicle recruitment in the mouse^36^. However, despite these differences, all these studies highlight that exposure to estrogenic EDCs, like DES, disrupt the regulation of early follicle transition and impact initial follicle recruitment, leading to altered folliculogenesis. Altered folliculogenesis is a mechanism that contributes to POI and early reproductive senescence, and therefore this observed phenotype could contribute to reduced fertility in DES descendants^14^. Alarmingly, we have shown that this altered folliculogenesis persists into the F2 generation. These results highlight the long-lasting impacts of EDCs and the importance in understanding the impact ancestral EDC exposure can have on females reproductive health.

Although no previous studies have investigated the multi- or trans-generational effect of DES on folliculogenesis, research has been conducted on the EDC DEHP. Pocar et al. investigated the effect of 0.05 and 5 mg/kg of DEHP exposure on follicle proportions in F1-F3 PND 42 female mice^37^. DEHP significantly reduced the number of primordial follicles and significantly increased the number of pre-antral follicles (primary and secondary follicles) in F1-F3 generations at both doses^37^. Similarly, prenatal exposure to 40 µg/kg of DEHP significantly reduces the percentage of primordial follicles and significantly increases the percentage of secondary follicles in both F1 and F2 females^38^. These studies align with the results presented here and together, demonstrate that developmental EDC exposure disrupts folliculogenesis and, importantly, these effects persist across generations.

Although the changes in folliculogenesis observed in our study are small, these results reflect the reproductive changes that are observed in human populations. Increases in the rates of fertility and sub-fertility have coincided with increased rates of reproductive disorders such as POI. Accelerated folliculogenesis appears to be an important factor contributing to reduced fertility in DES descendants, especially when coupled with other reproductive issues. Although DES is no longer in use in pregnancy, it is a valuable model for other estrogenic EDCs present in our environment and provides insight into the effects estrogenic EDCs can have on the development of disorders such as POI. POI is a multifactorial disease, with genetic, metabolic and environmental factors all playing a role in its onset. The prevalence of POI has gradually increased, yet the cause of POI onset remains unknown for the majority of patients^39^. Exposure to estrogenic EDCs may be a likely contributor to this increasing incidence, with EDC exposure exacerbating the onset of POI in women who are already at risk due to their genetic makeup. Therefore, there is a need for improved diagnosis and earlier detection of patients at risk of POI. Informing clinicians and patients about contributing factors such as exposure to EDCs will improve management and treatment of women with POI.

One mechanism that can lead to POI is increased follicle atresia. GCs are the largest population of cells in the follicle and are essential for maintaining the health of the oocyte. Any alterations to GCs, such as disrupted proliferation or development, can directly impact the oocyte, with apoptosis of the GCs leading to follicle atresia^40–42^. Cell culture studies have shown that treating GCs with BPA significantly decreases GC viability and increases GC apoptosis at a dose as low as 100 pM^43^. Our study found that F1 females had a higher percentage of Casp3 positive follicles than control females, although this increase was not significant. Moreover, although not significant, a slight increase in GC proliferation was observed at each generation. Similarly, a slight increase in both GC proliferation and follicle atresia, as determined by Ki27 and p27 markers respectively, is observed in lambs treated neonatally with either DES or BPA^35^. These subtle alterations to the GCs may contribute to altered folliculogenesis and therefore contribute to a POI phenotype. Furthermore, FOXL2 was used a marker for GC morphology in the current study as it is required to maintain a GC phenotype and mutations in *Foxl2 *have been associated with POI in mice^44,45^. However, DES exposure had no effect on FOXL2, with no qualitative differences observed between DES descendants and the control. Theca cells are another important lineage of somatic cells found in the ovarian follicle that are fundamental for steroidogenesis and follicular growth^46^. Theca cells are responsible for producing androgens, which are ultimately converted to estrogens by the GCs^47^. DES exposure may be able to disrupt theca development and in turn impact folliculogenesis. COUP-TFII is highly expressed in theca cells and was used as a marker for theca cell steroidogenesis in the current study^48^. However, no changes to COUP-TFII were observed after DES exposure. In rats, DES exposure alters both androgen production from the theca cells and estrogen production from the GCs^49^.Therefore, although no morphological differences in granulosa and theca cells were observed in our study, further studies would be required to isolate follicles following DES exposure and determine the individual hormonal production in granulosa and theca cells.

PCOS is one of the most common female reproductive disorders and is thus a major cause of female infertility. PCOS has been associated with EDC exposure in both humans and mice with prenatal exposure to a mixture of plastics significantly increasing the number of follicular cysts in F1 and F3 female mice^23^. Moreover, Brehm et al. also demonstrated that prenatal exposure to 750 mg/kg of DEHP significantly increases the number of ovarian cysts in mice^50^. In contrast, our study found that DES exposure did not increase the proportion of cystic follicles in any generation. While this suggests different modes of action of these EDCs, differences in the effects of these EDCs may also be due to the lower dose of DES used in our study. In order for PCOS to be diagnosed in females, two of the following three symptoms need to be observed; irregular ovulation, increased androgen levels and ovarian cysts^20^. As we only analysed the presence of follicular cysts in our study, future studies should assess androgen levels and ovulation patterns to further discern if DES has the potential to induce a PCOS phenotype and contribute to reduced fertility.

This is the first study that has investigated the transgenerational effect of DES on folliculogenesis. We have shown that a direct exposure to DES, either as a fetus or germ cells, accelerates follicle recruitment, a mechanism that can contribute to POI. This accelerated folliculogenesis can contribute to the reduced fertility observed in F1 and F2 DES females. However, altered folliculogenesis was not observed in F3 females, suggesting that DES does not impact ovarian function transgenerationally. Furthermore, DES did not increase the number of follicular cysts, suggesting that PCOS was not a contributing factor to reduced fertility in DES descendants. These results highlight the ability of estrogenic EDCs to disrupt normal ovarian function and contribute to the onset of POI and subsequent infertility.

## Methods

### Animals and treatments

C57BL/6 mice were raised in the animal facility at BioSciences 4 at the University of Melbourne under a 13 h light/11hr dark cycle at 22–23^o^C and fed a standard diet and tap water ad libitum. The University of Melbourne Ethics Committee, ethics number 1714163, approved all procedures. All experiments were performed in accordance with relevant guidelines and regulations. The study is reported in accordance with ARRIVE guidelines (https://arriveguidelines.org).

Eight-week-old female mice were mated to male mice and the presence of a vaginal plug was considered day 0 of gestation. These dams were considered the F0 generation. Dams were treated with 100 µg/kg body weight of DES every second day of gestation from day 9 to 17 inclusive to ensure exposure occurred throughout the window of fetal sexual differentiation and development^51^. DES (Abcam) was dissolved in 100% ethanol and diluted to 40 µg/ml in 1% ethanol in sterile phosphate buffered saline (PBS) to make up the stock solution. DES was administered via subcutaneous injection using a 26-gauge needle into the flank. This regimen represents one of the many delivery routes in which women received DES and the dose encapsulates the upper range of DES exposure pregnant women received between 1940 and 1970^3^. Control female mice (vehicle) were treated with 1% ethanol in phosphate buffered saline through subcutaneous injection in the same manner. Only one generation of mice was bred for the vehicle group, this data was used for the F1, F2 and F3 vehicle data.

At 8 weeks of age F1 female mice (exposed as fetus) were mated and housed with unexposed males for 2 weeks to generate the F2 generation. The F2 females were used to generate the F3 generation in the same manner.

### Tissue collection

Females were killed at either PND21 or 12 months of age for tissue collection. These two time points are the young and aged cohort respectively. In order to assess POI, females from each generation and control (*n*= 4–10) were sacrificed at PND 21 via cervical dislocation. Body weight and AGD were measured, with the standardised AGD for each animal being calculated as the AGD divided by the cubed root of body weight^29^. Ovaries were dissected, and one ovary was fixed in 4% PFA overnight at 4^o^C to be used for immunohistochemistry, the other ovary was fixed in Bouin’s solution overnight at 4^o^C for hematoxylin and eosin staining.

To determine the presence of follicular cysts, females from each generation and control (*n* = 3–6) were culled at 12 months of age via CO_2_ inhalation and cervical dislocation as a secondary kill method. Similarly, body weight and AGD were measured, and ovaries were collected and fixed in Bouin’s solution overnight at 4^o^C. All ovaries were processed and embedded in paraffin wax and serially sectioned at 7 μm.

### Follicle counts

**Fig. 10:**
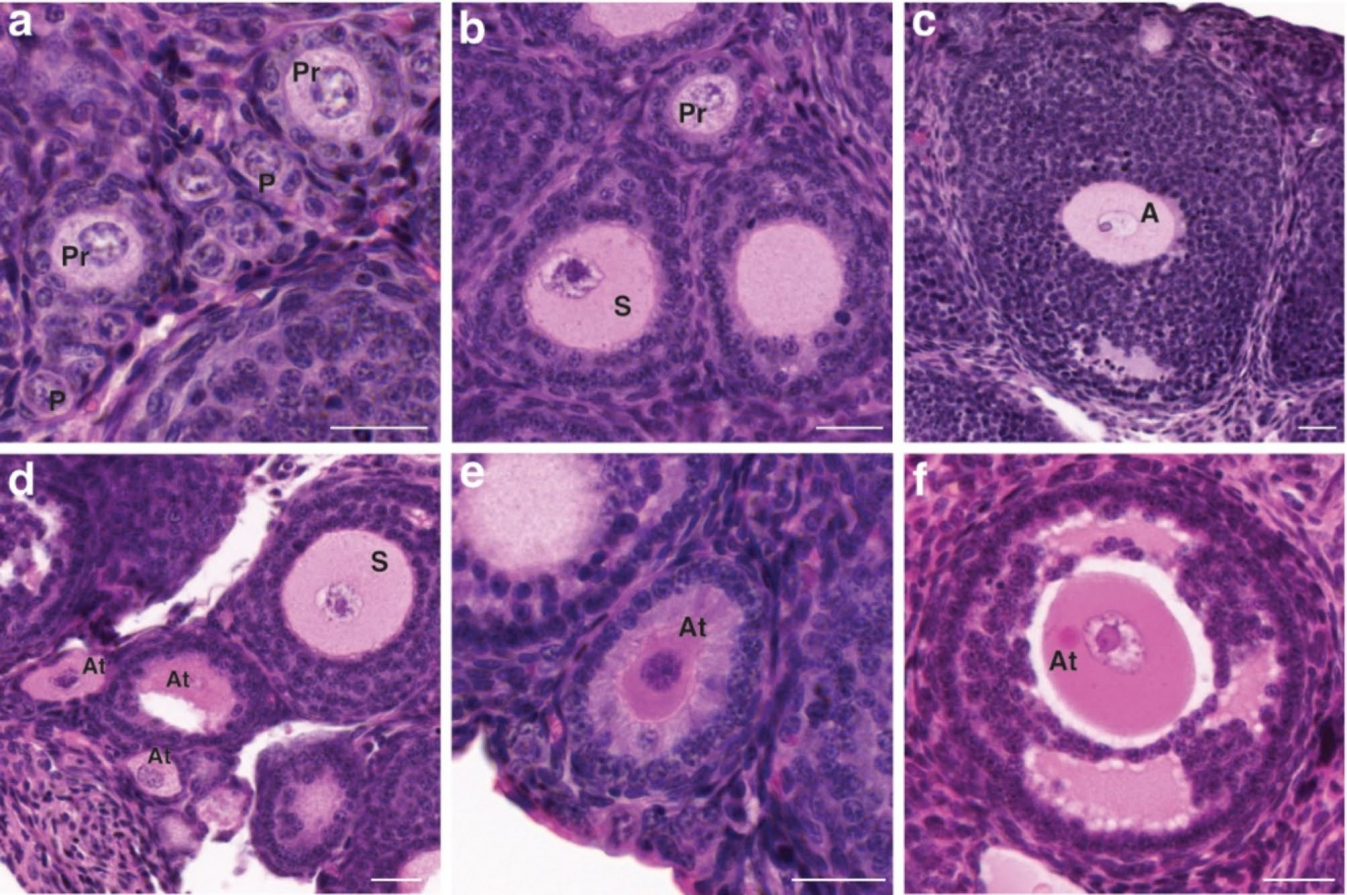
H&E stained ovarian sections representing various follicle types.** (a-c)** represent healthy follicle types, **(d-f)** represent atretic follicles. P, primordial follicles; Pr, primary follicles; S, secondary follicles; A, antral follicles; At, atretic follicles. Scale bar = 25 μm.

Bouin’s fixed ovaries from the young cohort were used for follicle counts. Ovarian sections were deparaffinised in histolene and re-hydrated in a decreasing gradient of ethanol. Slides were stained in hematoxylin for 7 min, decolorised with acid alcohol and Scott’s tap water and counterstained with eosin for up to 1 min. Slides were dehydrated and coverslipped and scanned using the 3D Histech Pannoramic ScanII Scanner. Follicle counts were carried out as described by Repouskou et al.^17^. Every 10th section was used to count primordial, primary, secondary, antral and atretic follicles. Follicles were classified as primordial if they had an oocyte surrounded by a single layer of squamous GCs and a developed zona pellucida; primary if the oocyte was surrounded by a unilaminar layer of cuboidal GCs; secondary if the oocyte was surrounded by more than one layer of GCs and a developing theca layer; antral if a fully grown oocyte was surrounded by many GC layers and a fluid filled antral space. To avoid double counting, only follicles with a visible nucleus were counted. Follicles were defined as atretic if two or more of the following features were present: degenerating oocyte, GC layer containing pyknotic nuclei, detached GC layer from the basement membrane or a broken basement membrane (Fig. 10)^17^.

Ovaries collected from the aged cohort were used to analyse the presence of follicular cysts. Every ovarian section was analysed for the presence of follicular cysts, with follicular cysts being defined as a fluid-filled structure lacking GCs and an oocyte and lined by one to four layers of cuboidal GCs^52^.

All analyses were conducted without knowledge of the treatment group or generation. Follicle types were represented as proportion of the total number of healthy follicles per individual rather than as raw numbers to account for variation in the total number of healthy follicles between each ovary.

### Immunohistochemistry

PFA fixed ovaries from the young cohort were used for immunohistochemistry analysis. For each mouse 10 sections from the mid portion of the ovary were analysed for cell proliferation and cell apoptosis with PCNA and Casp3 antibodies respectively. Sections were dewaxed in histolene and rehydrated in a decreasing gradient of ethanol followed by heat-induced epitope retrieval using 10 mM sodium citrate pH 6.0 at below boiling for 8 min. Sections were cooled to room temperature for 30 min and endogenous peroxidase activity was blocked using 3% H_2_O_2_ for 5 min. Non-specific sites were blocked with 10% normal horse serum and 3% BSA in TBS for 1 h. Sections were then incubated with PCNA primary antibody (1:3600 dilution, Cell Signalling, D3H8P), Casp3 primary antibody (1:400 dilution, Cell Signalling, 5A1E), control rabbit IgG or negative control in 0.1% BSA in TBS overnight at 4^o^C. Sections were incubated with biotinylated horse anti rabbit secondary antibody (1:1000 dilution, Vector Laboratories, BA-1100) for 1 h at room temperature followed by 30 min incubation with avidin-biotin complex with horseradish peroxidase. Sections were developed and visualised using DAB. Sections were counterstained with haematoxylin for 10 min, dehydrated, cleared with histolene and coverslipped. The 3D Histech Pannoramic ScanII Scanner was used to scan all slides. QuPath software was used to determine the number of positive DAB GCs as a proportion of the total number of GCs in each follicle. To avoid double counting only follicles containing a nucleus were analysed. A follicle was considered to be positive for Casp3 if 5% or more of the granulosa cells were Casp3 positive. The number of positive PCNA stained cells were calculated as a percentage of the total number of GCs within a follicle.

### Immunofluorescence

IF was used to analyse granulosa and theca cell morphology in the young cohort. Ovarian sections that had been fixed in PFA were used for IF analysis, one slide from the middle section of the ovary was taken from each animal. Sections were dewaxed in histolone and rehydrated in a decreasing gradient of ethanol. Sections were submerged in sodium citrate buffer and heated to below boiling for 10 min, after which they were left to cool to room temperature for 1 h. Sections were blocked with 10% normal goat serum and 3% BSA in PBS and 0.1% triton for 1 h at room temperature. Sections were then incubated with FOXL2 primary antibody (1:300 dilution, Dagmar Wilhelm, University of Melbourne) to mark GCs and COUP-TFII primary antibody (1:200 dilution, Perseus Proteomics, PP-H7141-00) to mark theca cells overnight at 4^o^C. Sections were incubated with secondary antibodies Alexa Fluor 488 donkey anti-rabbit (Invitrogen, A-21206) and Alexa Flour 555 donkey anti-mouse (Invitrogen, A31570) overnight at 4^o^C. Sections were counterstained with DAPI to visualise cell nuclei. Negative controls were sections where the primary antibody incubation was omitted. Sections were mounted with fluorescent mounting media (Dako) coverslipped and visualised using a confocal microscope (Nikon A1R).

### Statistical analysis

Where data had equal variance and normal distribution, a one-way analysis of variance was performed, and when a significant *p* value was obtained, a Tukey’s test was used in the post hoc analysis. When data did not have either equal variance or normal distribution, a Wilcoxon pairwise test was used followed by a post hoc Kruskal-Wallis rank sum test. A *p* value of < 0.05 was considered significant.

## Data Availability

The datasets used and/or analysed during the current study available from the corresponding author on reasonable request.
